# Genomic and clinical characteristics of *MET* exon14 alterations in a large cohort of Chinese cancer patients revealed distinct features and a novel resistance mechanism for crizotinib

**DOI:** 10.7150/jca.49391

**Published:** 2021-01-01

**Authors:** Tianli Cheng, Zhongping Gu, Danni Song, Sisi Liu, Xiaoling Tong, Xue Wu, Zhifeng Lin, Wei Hong

**Affiliations:** 1 Thoracic Medicine Department 1, Hunan Cancer Hospital, Affiliated Tumor Hospital of Xiangya Medical School of Central South University, Changsha, Hunan, China.; 2 Department of Thoracic Surgery, The Second Affiliated Hospital, Air Force Military Medical University, Xi'an, China.; 3 Department of Research and Development, Nanjing Geneseeq Technology Inc., Nanjing, Jiangsu, China.; 4 Translational Medicine Research Institute, Geneseeq Technology Inc., Toronto, Ontario, Canada.; 5 Department of Thoracic Surgery, Shanghai General Hospital, Shanghai, China.; 6 Department of Medical Oncology, Cancer Hospital of the University of Chinese Academy of Sciences, Zhejiang Cancer Hospital, Hangzhou, China.; 7 Institute of Cancer and Basic Medicine (IBMC), Chinese Academy of Sciences, Hangzhou, China.

**Keywords:** *MET* exon 14 alterations, lung cancer, next-generation sequencing, crizotinib-resistant mutation

## Abstract

**Background:** Alterations in *MET* exon 14 (*MET*ex14) and its flanking intronic regions have been identified in a variety of cancers. Patients with *MET*ex14 alterations often benefit from MET inhibitors such as crizotinib. Given the unique mutation profiles of Chinese lung cancer patients, it is necessary to investigate the prevalence of *MET*ex14 alterations in a large cohort of cancer patients.

**Patients and methods:** Cases carrying *MET*ex14 alterations were screened from 26,391 Chinese cancer patients by next-generation sequencing (NGS), and the clinicopathologic and molecular characteristics were reviewed.

**Results:** Compared to Western population (~3%), the frequency of *MET*ex14 alterations is much lower in Chinese cancer patients (0.7%, n=184) and lung cancer patients (1.1%, n=175). Seventy-eight distinct *MET*ex14 alterations, including several novel alteration types were detected. Concurrent *MET* copy gain and non-exon14 *MET* mutations were also found. *EGFR* copy gain (11%) and mutations (8%), *KRAS* (5%) and *PIK3CA* (5%), appeared in a mutually exclusive pattern. Female patients contain much less *TP53* mutations than male patients (65% vs. 24%, FDR = 0.01). Co-amplification of *CDK4* and *MDM2*, *CDK6* and *EGFR* were identified, which indicated cell cycle dysregulation and *EGFR* alteration are important co-occurring features in patients with *MET*ex 14 alteration. Of 9 tissue specimens having PD-L1 immunohistochemistry (IHC) results, 5 of them (55.5%) were found PD-L1 positive, which is comparable to other types of tumor. In 14 crizotinib-treated patients, the median progression free survival (mPFS) was 7 months. Upon resistance to crizotinib, two patients acquired secondary mutations in *MET* and one patient acquired *BRAF* p.K601E that can be a novel resistance mechanism.

**Conclusion:** Chinese cancer patients have a relatively lower frequency of *MET*ex14 alterations compared to Western patients. Patients with *MET*ex14 alterations showed distinct molecular characteristics and the representative case study showed responses to MET tyrosine kinase inhibitor (TKI).

## Introduction

The hepatocyte growth factor (HGF) receptor, encoded by the* MET* oncogene, is a receptor tyrosine kinase that activates a wide range of cellular signaling pathways to mediate cell proliferation, survival and motility, and subsequently induces cancer development and progression 1, 2. Pathologic activation of *MET* were frequently caused by point mutations in tyrosine kinase domain, gene copy gains and protein overexpression 3, 4, and less frequently, by alterations affected the splicing of exon14, which resulted in *MET* exon14 (*MET*ex14) skipping after translation. Besides, loss of p.Y1003 in exon 14, a critical binding site for Cbl (an E3 ubiquitin ligase) will also disrupt MET ubiquitination and degradation, leading to the overactivation of MET signaling 5, 6. *MET*ex14 skipping has been identified in a wide variety of human cancers with an incidence of around 3% in all cancer types 7. The alterations are highly diversified, including point mutations at splicing sites, in-frame deletion of intronic region around splicing sites at various lengths and the large fragment deletion to remove the entire exon 14 8, 9.

Preclinical and clinical evidences suggest that tumors with *MET*ex14 are sensitive to small molecule tyrosine kinase inhibitors (TKIs), including non-selective inhibitor crizotinib 10, and several selective inhibitors, such as tepotinib, savilitinib and capmatinib 11-13. In 2020, FDA approved the first targeted therapy capmatinib for *MET*ex14-positive patients with locally advanced or metastatic non-small cell lung cancer (NSCLC).

With the advanced parallel sequencing technologies, it becomes much easier to screen for novel alterations in *MET*ex14 and comprehensively analyze the concurrent genomic alterations, thereby expand the number of druggable patients and uncover the intrinsic or acquired resistant mechanisms to TKI treatment. In this study, we retrospectively screened genomic data of 26,391 Chinese cancer patients and identified 184 cases with *MET*ex14 alterations in 10 different cancer types, among which 175 were lung cancer patients. Seventy-eight unique *MET*ex14 alterations were identified, including novel alterations c.2888-90_2899del, c.2888-55_2928del. comprehensively, genomic profiling also revealed concurrent and exclusive gene alterations in these patients. Clinical responses were observed in 14 patients who received crizotinib treatment and potential drug-resistance mechanisms were analyzed.

## Materials and methods

### Patients and samples

Genomic profiling results of 26,391 malignant tumors were screened and only samples with alterations in *MET* exon 14 or intron 13 and 14 that could potentially cause *MET* exon 14 skipping or the loss of MET p.Y1003 residue were analyzed. Genomic profiling of these samples was performed on formalin-fixed paraffin-embedded (FFPE) tumor/plasma biopsy specimens that were obtained from patients signed written informed consent.

### Next-generation sequencing (NGS)

DNA extraction and sequencing library were prepared according to the protocols described previously 14, 15. To be specific, 104 and 80 cancer samples were tested with 139-gene panel and 425-gene panel respectively, both of which cover the whole *MET* exon 14 and the adjacent intron regions, and all essential lung-cancer related genes. All samples were sequenced in a Clinical Laboratory Improvement Amendments (CLIA)- and College of American Pathologists (CAP)-certified genomic testing facility (Nanjing Geneseeq Technology Inc., Nanjing, China). Different types of genetic alterations were called using an internally-validated bioinformatics analysis pipeline 16. Clinical information, including age at diagnosis, sex, disease stage, and treatment history was extracted from the medical records provided by physicians during the service order. Informed written consent was acquired from each patient at the time of sample submission. The study methodologies conformed to the standards set by the Declaration of Helsinki and was approved by the ethics committee.

### Immunohistochemistry (IHC) staining of PD-L1

Eleven tissue biopsies of this study were performed IHC staining of PD-L1 expression with 22C3 anti-PD-L1 antibody (Dako) according to the protocol reported previously 17. PD-L1 IHC was evaluated by a pathologist based on the tumor proportion score (TPS) with membranous and/or cytoplasmic staining, and divided into three groups: no-expression (<1% of tumor cells), low expression (1%-49%) and high expression (≥50%).

### Data analysis and statistics

All statistical tests were conducted in R version 3.6.1. The concurrent mutations and exclusive/concurrent analysis were conducted with the SomaticInteraction function in Maftools package of R 18 and a *p*-value below 0.05 was considered as significant.

## Results

### Various *MET*ex14 alterations were identified in different cancer types

*MET*ex14 alterations were identified in 184 of 26,391 (0.7%) patients from 9 cancer types (**Supplementary Fig. S1**), including lung cancer (1.1%, 175/18112, **Table 1**), neuroendocrine tumor (NET, 1/85), bladder cancer (BLCA, 1/68), breast cancer (BRCA, 1/3639), colorectal cancer (CRC, 2/2551), pancreatic cancer (PACA, 1/661), esophageal cancer (ESCA, 1/549), cholangiocarcinoma (CHOL, 1/547) and melanoma (1/179). The frequency of *MET*ex14 alterations in Chinese patients is lower than 3% in Western countries 19. In lung cancer patients with *MET*ex14 alterations, 79.4% (139/175) were non-small-cell lung cancer (NSCLC), including adenocarcinoma (ADC, 114/139, 82.0%), adenosquamous carcinoma (ASC, 9/139, 6.5%), squamous cell carcinoma (SCC, 8/139, 5.8%), large cell carcinoma (LCC, 1/139, 0.7%) and subtype-undetermined NSCLC (7/326, 2.1%) (**Table 1**). Thirty-six patients (36/175) were histologically undetermined.

**Figure 1:**
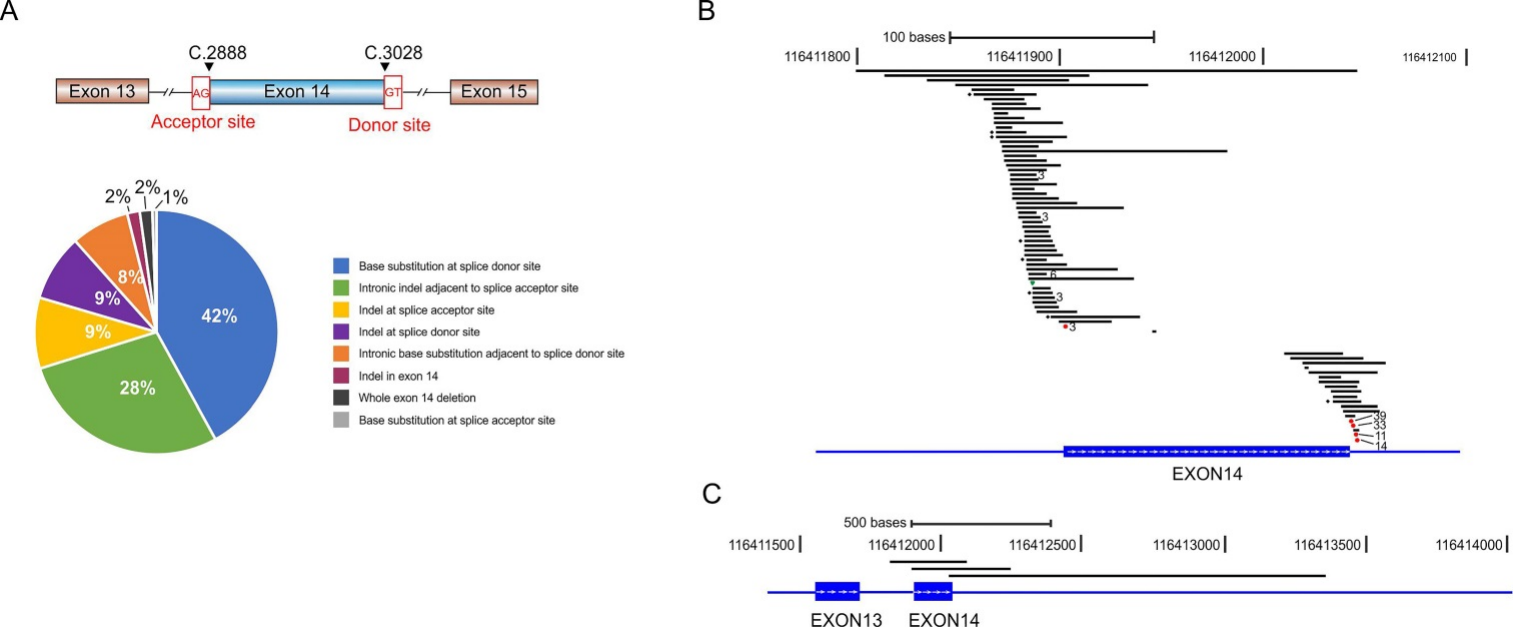
** Various *MET*ex14 alterations and their schematic locations around exon 14.** A) Pie chart shows the frequency of different *MET*ex14 alterations that were grouped by the locations and mutation types. B-C) Schematic view of each *MET*ex14 alteration on human genome build GRCh37/hg19. The frequency of each *MET*ex14 are indicated with * for two and the number of cases for greater than two, as labeled behind.

*MET*ex14 alterations comprise base substitution, insertion, and large fragment deletion spanning the entire or partial region of exon 14. We identified 78 distinct *MET*ex14 alterations and classified them into 8 subgroups according to the alteration types and locations (**Fig. 1A** and** Supplementary Table S1**). Base substitutions at the splicing donor sites were the most common alterations (42% of all *MET*ex14 alterations), represented by c.3082G > T/A/C (39/184, 21.2%), c.3082 + 1G > C/A/T (33/184, 17.9%), c.3082 + 2T > A/C/G (11/184, 6.0%) and c.3082 + 3A > T/C/G (14/184, 7.6%) (**Fig. 1B, Supplementary Table S1**). We also identified a great amount of indel alterations spanning the ~50 bp intronic upstream region of the splice acceptor site (28%), and base substitutions immediately adjacent to the splice donor site (8%, **Fig. 1A and 1B**). Three cases have large fragment deletion that removed the entire *MET*ex14 (**Fig. 1C**). There are also three insertion/deletions (indels) within *MET*ex14 that directly disrupt the ubiquitin ligase site (p.Y1003), including *MET* p.E999_P1008del, p.T1006_P1008del and p.D1002_Y1003del (**Supplementary Table S1**). Additionally, three patients harbored two different *MET*ex14 alteration types respectively (**Supplementary Table S2**).

### Clinical characteristics and genomic profiling of lung cancer with *MET*ex14 alterations

The prevalence of *MET*ex14 alterations was much higher in ASC (6.25%) than other subtypes, including LCC (2.7%), ADC (1.0%) and SCC (0.7%) (**Table 1**). The median age of lung cancer patients with *MET*ex14 was 68 (range from 39 to 94), and females and males take up 48.6% (n=86) and 47.4% (n=84), respectively.

**Figure 2:**
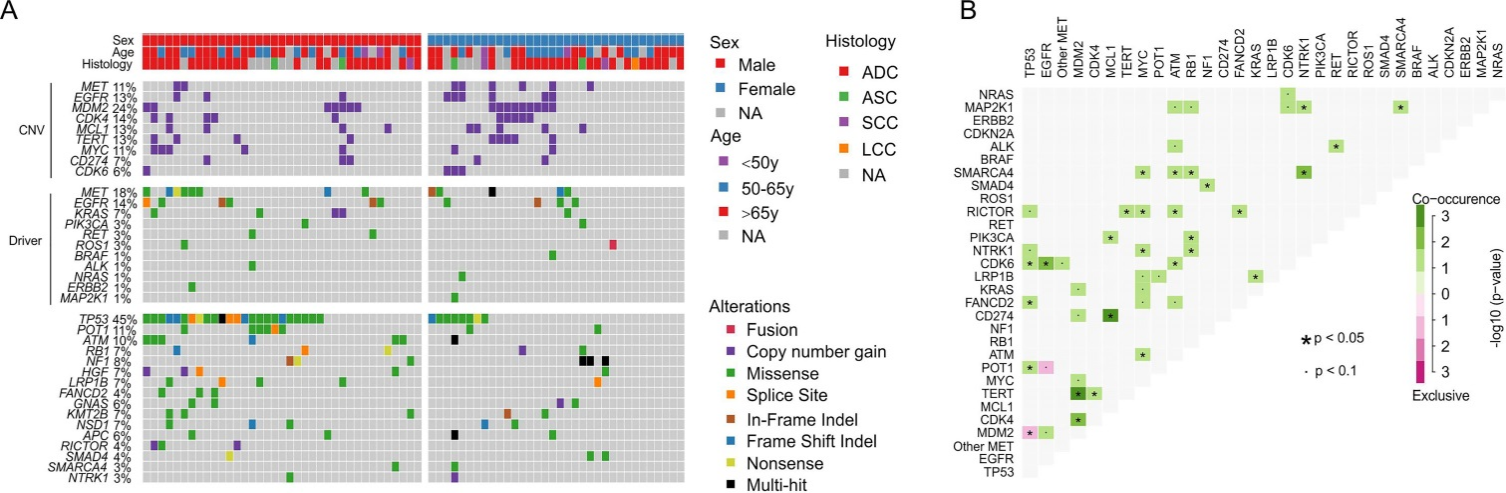
** Comprehensive genomic profiling of 71 lung cancer patients sequenced by 425-gene panel.** A) Co-mutation plot of clinically relevant and frequently altered genes. B) Concurrence and exclusive analysis of gene alterations. A p-value < 0.05 is considered statistically significant.

Genomic profiling of 175 lung cancer tumors revealed the high prevalence of *TP53* alterations (43%) and relatively low co-occurrence of other cancer driver alterations, such as *EGFR* alterations (19%, **Supplementary Fig. S2**), which is much less than 40~60% in Asian lung cancer patients 20, 21. The secondary *MET* alterations were detected in 15% of patients and 11% of them have *MET* copy gain (**Supplementary Fig. S2**). For 71 cases that were examined with the 425-gene panel, mutation frequencies of different genes were comparable between females and males, except that male patients were significantly more enriched with *TP53* alterations than females (65% vs. 24%, FDR = 0.01, **Fig. 2A**). We observed a subpopulation that were prevalent with gene copy gains in *MDM2* (24%), *CDK4* (14%), *MCL1* (13%), *TERT* (13%), *MYC* (11%) and *CDK6* (6%), and showed a tendency of co-occurrence, e.g. *MDM2* with *TERT* and *CDK4* (*p* < 0.05, **Fig. 2**). Moreover, *CD274* (PD-L1), the biomarker for immunotherapy, was also found to be amplified in 7% of cases and mostly co-existed with *MCL1* copy gain (*p* < 0.05, **Fig. 2B**). *EGFR* copy gain and mutations were also identified in 13% and 15% patients, which is higher than previously reported frequency in Western lung cancer patients 9. *POT1*, a component that binds and protects telomere 22, was mutated in 11% of cases and likely to co-occur with *TP53* alterations (*p* < 0.05, **Fig. 2B**). The only significantly exclusive gene pair was *TP53* and *MDM2*, a negative regulator of TP53 (*p* < 0.05). These data suggested the dysregulation of cell cycle and EGFR signaling pathway in tumorigenesis of patients with *MET*ex 14 alteration.

Comparatively, gene alterations in 9 non-lung cancer cases revealed a different mutational spectrum, including a much higher occurrence of *TP53* alterations (89%), and the absence of *MDM2/CDK4* amplification. Moreover, *KRAS* mutations and non-*MET*ex14 alterations were found in 44% of cases (**Supplementary Fig. S3**), suggesting the companion mutations might be different across cancer types.

### Potential treatment strategies for patients with *MET*ex1*4* alterations

**Figure 3:**
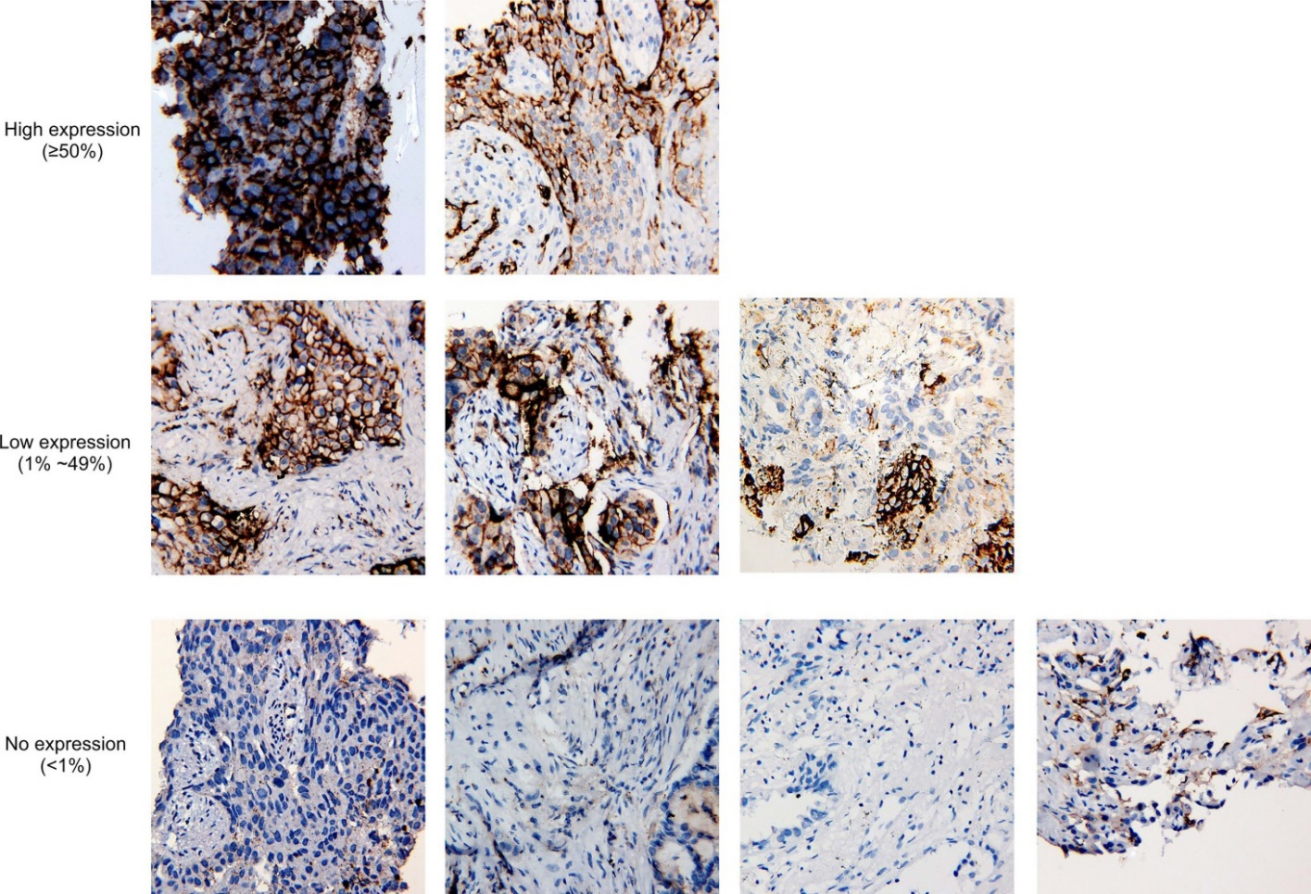
** Tumor proportion score of PD-L1 in nine cases with PD-L1 immunohistochemistry staining results available.** At 20x magnification, no expression of PD-L1 is defined as <1% of tumor cells in the sample, while low expression and high expression are defined by 1%-49% of tumor cells and ≥50% of tumor cells, respectively.

It has been reported that *MET* amplification was associated with increased PD-L1 expression 23. For 9 tissue biopsies having available PD-L1 IHC staining results, 5 samples were PD-L1 positive, including two high-expression samples and three low-expression samples (**Fig. 3**), and the frequency is comparable to the overall positive ratio in lung cancer 24, which shows no correlation between *MET*ex14 alteration and PD-L1 overexpression.

**Figure 4:**
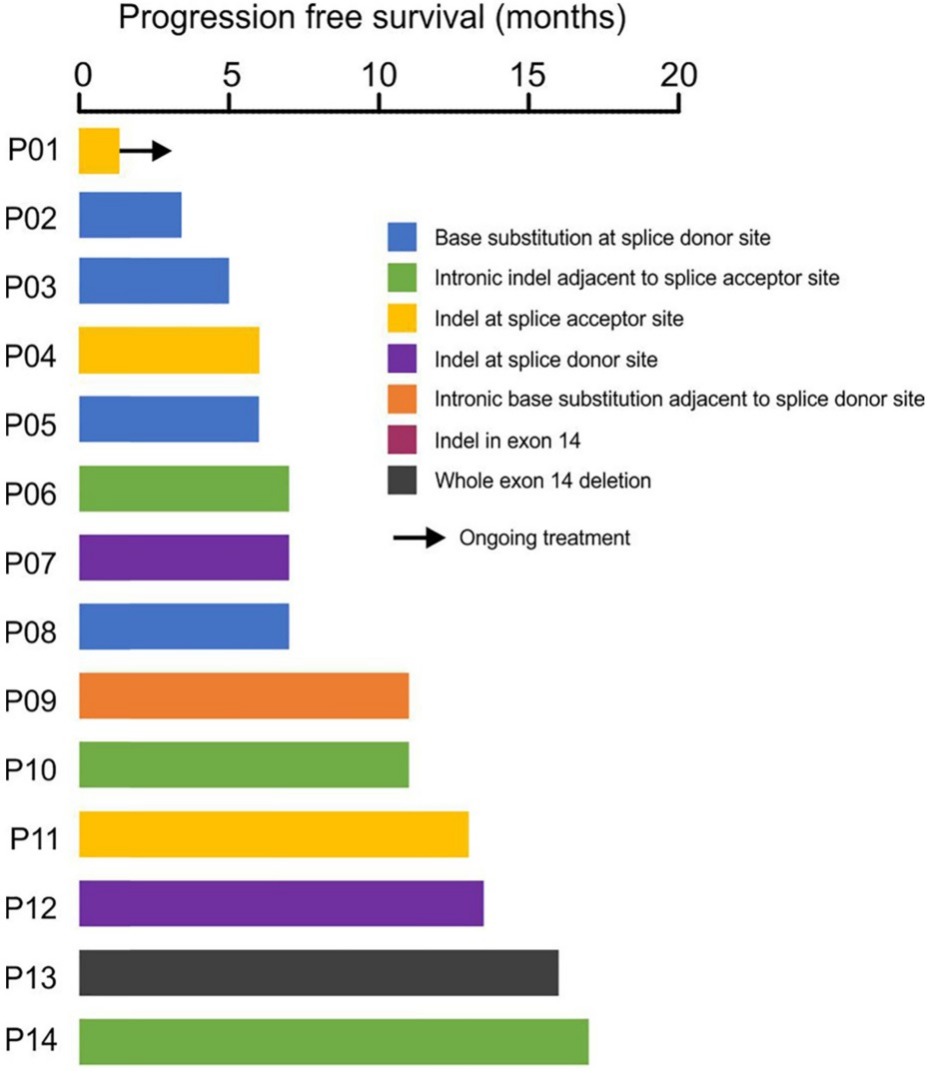
** Response of crizotinib-treatment in 14 patients with *MET*ex14 skipping.** The x-axis showed the patient ID and the y-axis showed the progression-free survival (PFS) in months.

Clinical treatment records showed that 14 patients have received crizotinib treatment, and the median progress-free survival (mPFS) was 7 months with the longest PFS of 17 months (**Fig. 4**). Four patients (P06, P09, P10, P14) with *MET*ex*14* alterations in the intronic region adjacent to splice acceptor site showed durable response to crizotinib treatment, suggesting the loss of exon 14 by these alterations (**Fig. 4**). Acquired secondary mutations, including *MET* p.Y1230N, p.D1228N and *BRAF* p.K601E were identified in the available post-treatment tumors from three patients (P05, P12, P14, **Fig. 4, Supplementary Table S3**).

Patient P05 was a 60-year-old female diagnosed with advanced lung adenocarcinoma, and was carrying a *MET*ex14 skipping mutation (c.3028+1G>C). After 4 months of crizotinib treatment, the disease progressed and a *BRAF* p.K601E mutation was identified. *BRAF* p.K601E is an activating mutation occurred in 0.15% of all NSCLC patients and showed low sensitivity to BRAF-targeted therapy in clinical studies 25-29, which could mediate crizotinib resistance in treatment.

Patient P12 was a 65-year-old female diagnosed with stage IV lung adenocarcinoma and underwent surgery after diagnosis. Disease was relapsed with bone metastasis after 12 months and a *MET*ex14 skipping alteration (c.2888-88_2901del) was detected. The patient was subjected to crizotinib treatment for 13 months, and a secondary mutation *MET* p.Y1230N was identified in plasma sample after resistance.

Patient P14 was a 61-year-old female and diagnosed with stage IV non-small cell lung cancer. She harbored *MET* c.2888-22_2888-8del and was subjected to crizotinib for 17 months. Post-treatment sample was detected with *MET* p.D1228N mutation.

## Discussion

This is the largest cohort study of *MET*ex14 alterations in a variety of cancer types. The incidence of *MET*ex14 alterations in Chinese lung cancer patients was 1.1%, which is lower than 3% in Western countries 8, 9, but its frequency in lung adenosquamous carcinoma is higher (6.5% vs 2.8%) 8, suggesting different mutation frequency of *MET*ex14 between Asian and Western lung cancer patients. We also identified an LCC patient carried *MET*ex14 alteration, which has not been reported. There is no significant difference of sex in our data, differing from the Western people (male vs. female=39.6% vs 60.4%) 9.

Seventy-eight unique *MET*ex14 alterations were identified and some of them have not been reported before. Aside from base substitutions of splice acceptor and donor sites 9, 19, insertion/deletions within/across intron13, exon 14 and intron 15 at different lengths were observed. In our data, the frequency of patients with deletion of entire *MET*ex14 is higher than the Western countries (2% vs. 0.7%) 8, 9 . A number of patients were also carrying other driver mutations, such as copy gain of *MET* and *EGFR*, non-exon14 *MET* mutations and *EGFR* mutations, suggesting that other driving forces in addition to *MET*ex14 alteration might be required for tumor initiation and evolvement.

Similar as previous reports, we observed copy gain of *MDM2*, *CDK4*, *TERT*, *MYC* and *MCL1* at different frequencies, but with a tendency of co-occurring. MDM2 is a negative regulator of TP53 by mediating TP53's degradation 30, and its amplification has been identified in a variety of cancers at a frequency of ~3%, which might have a potential role in treatment resistance of prostate cancer, neuroblastoma and lung cancer 31-33. Here, we found that *MDM2* amplification is likely to be exclusive with *TP53* alterations, possibly because the functional overlap between these two genes. We also observed co-occurrent of copy number gain of *MDM2* with cell cycle-dependent kinase *CDK4*. *MDM2* and *CDK4* are frequently co-amplified in NSCLC 34 and sarcomas, and play crucial roles in tumorigenesis via increasing cell growth and migration 35. There are statistically significant associations between copy number gain of *EGFR* and *CDK6*, which is consistent with a previous study in glioma 36. These data suggest cell cycle dysregulation and *EGFR* alteration are important co-occurring features in patients with *MET*ex 14 alteration. Since inhibitors to cell cycle are approved or clinically tested for therapy, patients with *MET*ex 14 alteration may have better outcome from treatment targeting both MET and cell cycle. Studies have shown that the down-regulation of *CD274* (PD-L1) and the apoptosis gene *MCL1* is synchronized 37. Our data shows here a co-occurrence of copy number amplification between* PDL1* and *MCL1*, suggesting that there may be some positive regulatory relationship between the two genes.

Lastly, in 14 patients who were treated with crizotinib, the PFS varied from 4 months to as long as 17 months. Two patients acquired secondary *MET* mutations, including *MET* p.Y1230N and p.D1228N, which have been reported as the potential resistance mechanisms after treating *MET*ex14 skipping with crizotinib 38-40. Additionally, acquired *BRAF* p.K601E was found in another crizotinib-resistance patient with only 4-month PFS. Similar to BRAF p.V600E, BRAF p.K601E is also an activating mutation of BRAF resulting in continuous activation of MEK/ERK signaling pathway 41, 42, therefore can be considered as a bypass resistance mechanism.

## Conclusion

The incidence of *MET*ex14 skipping was lower in Chinese cancer patients than Western cancer patients, but its prevalence in lung adenocarcinoma is higher than Western patients. The alteration is highly diversified and deep into the intron region. Therefore, it requires precaution when choosing the right test for it. We observed treatment efficacy of crizotinib in some patients and reported potential resistance mechanisms in a few cases. Several MET inhibitors, including crizotinib, are currently under evaluation for the treatment of NSCLC patients with METex14 skipping and capmatinib was recently approved by FDA to treat this alteration in NSCLC. Along with the increased use of these small molecule TKIs in treatment, more acquired resistance mechanisms will be investigated.

## Supplementary Material

Supplementary figures and tables.

## Figures and Tables

**Table 1:** Clinical and molecular characteristics of lung cancer patients harboring METex14 skipping

	Total lung cancer	NSCLC					Lung cancer with unknown histological subtype
		ADC	SCC	ASC	LCC	Subtype-undetermined NSCLC	
Cases with *MET* Exon14 skipping, n/N (%) ^a^	175/18112 (1.1)	114/11208 (1.0)	8/1133 (0.7)	9/144 (6.25)	1/37 (2.7)	7/326 (2.1)	36/4851 (0.7)
Median age (range), y	67 (39-94)	68 (43-94)	74 (55-81)	70 (39-83)	61 (61-61)	64 (56-73)	74 (49-87)
**Sex, n (%)**							
Male	71 (51.1)	58 (50.9)	4 (50.0)	5 (55.6)	0	4 (57.1)	15 (41.7)
Female	68 (48.9)	56 (49.1)	4 (50.0)	4 (44.4)	1 (100.0)	3 (42.9)	21 (58.3)
**Stage, n (%)**							
I-II	9(6.5)	8(7.0)	0	1 (11.1)	0	0	1 (2.7)
III	7 (5.0)	5 (4.4)	0	1 (11.1)	0	1 (14.3)	0
IV	29 (20.9)	24 (21.1)	1 (12.5)	2 (22.2)	0	3 (42.9)	4 (11.1)
Unknown	94 (67.6)	77 (67.5)	7 (87.5)	5 (55.5)	1 (100.0)	3 (42.9)	31 (86.1)
**Smoking history, n (%)**							
Yes	5 (3.6)	3 (2.6)	0	1 (11.1)	0	1 (14.3)	2 (5.6)
No	17 (12.2)	15 (13.2)	0	1 (11.1)	0	1 (14.3)	3 (8.3)
Unknown	117 (84.2)	96 (84.2)	8 (100.0)	7 (77.7)	1 (100.0)	5 (71.4)	31 (86.1)

a, n/N represents the occurrence of MET exon 14 skipping and the total number of patients in each subcategory.
