# Supplementary material for: Genomic and clinical characteristics of MET exon14 alterations in a large cohort of Chinese cancer patients revealed distinct features and a novel resistance mechanism for crizotinib
Source: J Cancer. 2021 Jan 1;12(3):644–51. doi: 10.7150/jca.49391 (PMC7778531; doi:10.7150/jca.49391)
Supplement: Supplementary file 1 — Supplementary figures and tables. [file jcav12p0644s1.pdf]

Supplementary Information

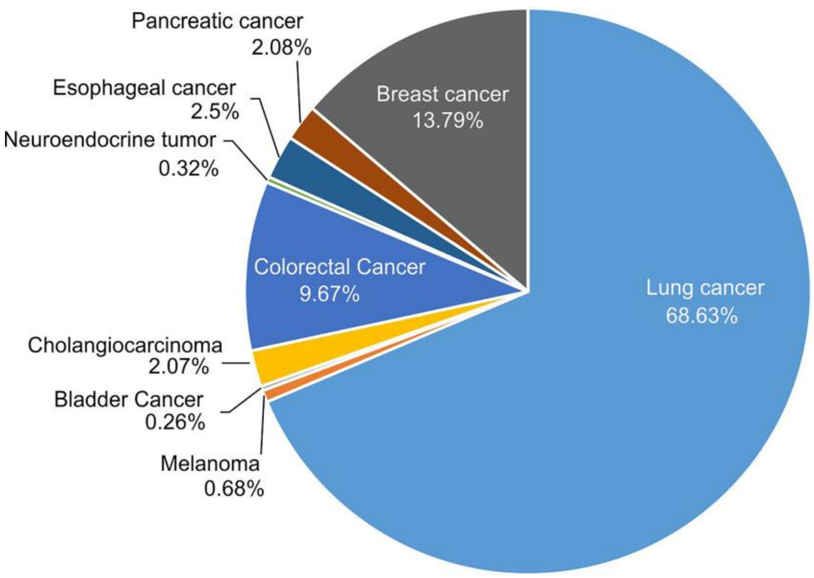

Supplementary Figure 1. Cancer type distribution of 26,391 screened cancer patients (A) and the mutation spectrum of nine non-lung cancer patients (B).

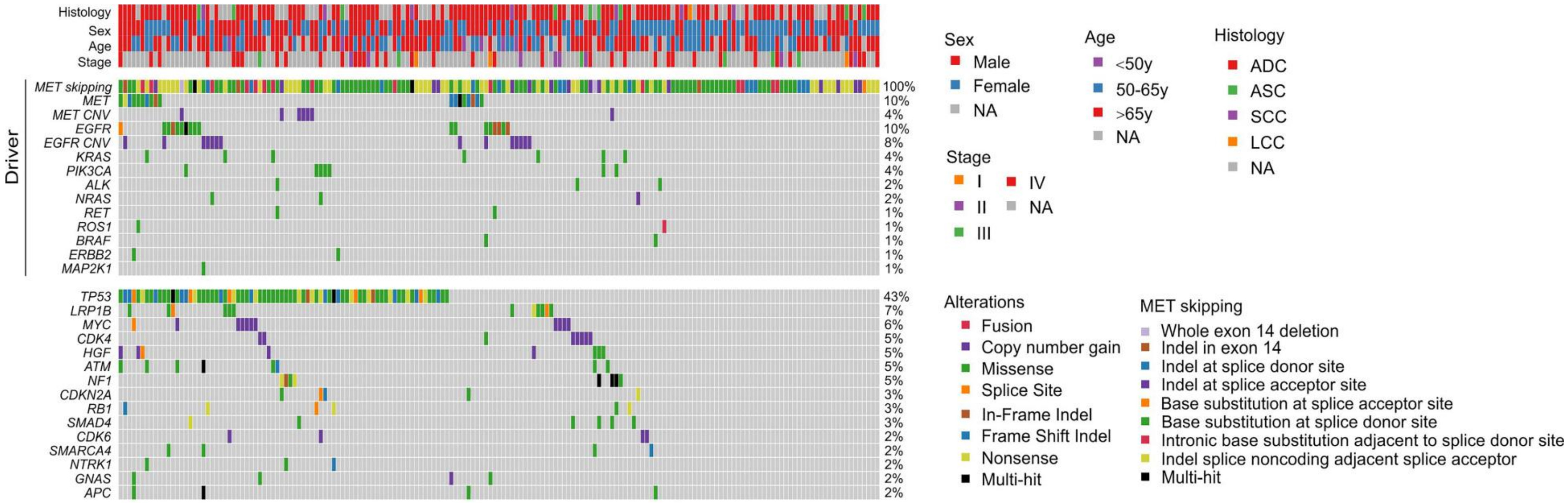

Supplementary Figure 2. Co-mutational plot of 175 lung cancer patients using the shared genes between 139- and 425-gene panels. All mutations in driver genes are plotted, and for non-drivers, only gene alterations with a frequency of greater than 2% are displayed.

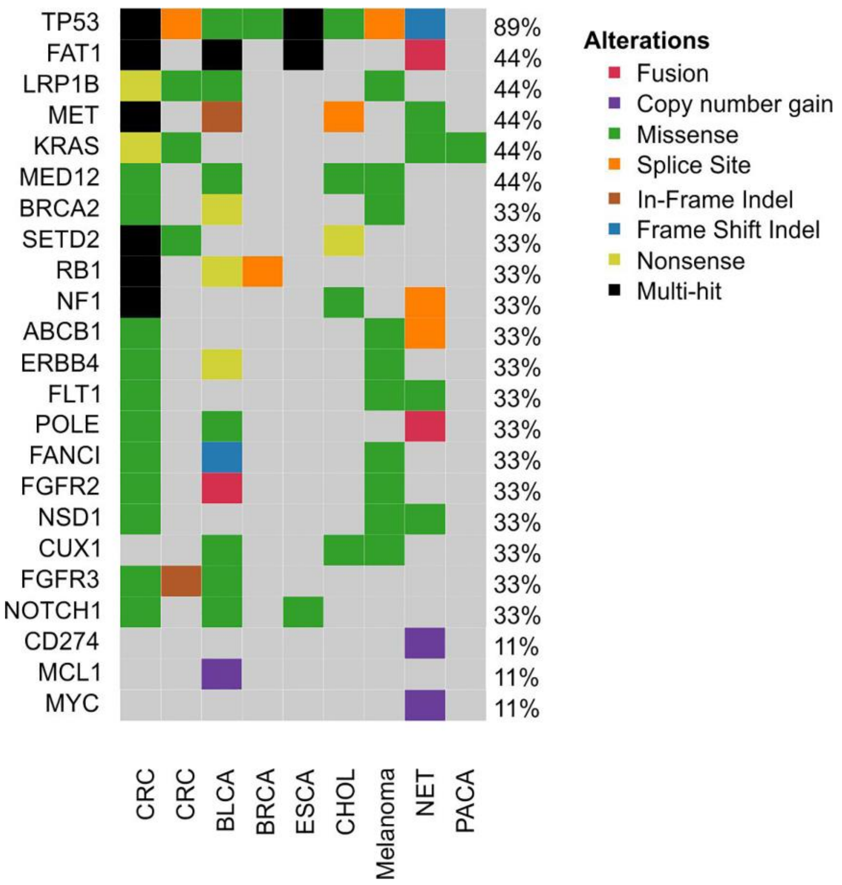

Supplementary Figure 3. Co-mutational plot of 9 non-lung cancer patients

Supplementary Table 1.

Gene	Exon/Intron Num.	Alternations	Chromosome Start Point	Chromosome End Point	Reference Sequence	Alternation Sequence
MET	exon14	c.2887+91_3028+3del	chr7:116411799	chr7:116412046	TATATGTAGTCCATAAAACCCATGAGTTCTGGGCACTGGGTCAAAGTCTCCTGGGGCCCATGATAGCCGTCTTTAACAAGCTCTTTCTTTCTCTCTGTTTTAAGATCTGGGCAGTGAATTAGTTCGCTACGATGCAAGAGTACACACTCCTCATTTGGATAGGCTTGTAAGTGCCCGAAGTGTAAGCCCAACTACAGAAATGGTTTCAAATGAATCTGTAGACTACCGAGCTACTTTTCCAGAAGGTA	-
MET	exon14	c.2888-90_2899del	chr7:116411813	chr7:116411914	AAAACCCATGAGTTCTGGGCACTGGGTCAAAGTCTCCTGGGGCCCATGATAGCCGTCTTTAACAAGCTCTTTCTTTCTCTCTGTTTTAAGATCTGGGCAGTG	-
MET	exon14	c.2888-69_2889del	chr7:116411834	chr7:116411904	CTGGGTCAAAGTCTCCTGGGGCCCATGATAGCCGTCTTTAACAAGCTCTTTCTTTCTCTCTGTTTTAAGAT	-
MET	exon14	c.2888-55_2928 del&insA	chr7:116411848	chr7:116411943	CCTGGGGCCCATGATAGCCGTCTTTAACAAGCTCTTTCTTTCTCTCTGTTTTAAGATCTGGGCAGTGAATTAGTTCGCTACGATGCAAGAGTACAC	-
MET	intron13	c.2888-47_2888-26del&insGGT	chr7:116411856	chr7:116411877	CCATGATAGCCGTCTTTAACAA	GGT
MET	intron13	c.2888-46_2888-15del	chr7:116411857	chr7:116411888	CATGATAGCCGTCTTTAACAAGCTCTTTCTTT	-
MET	intron13	c.2888-41_2888-21del	chr7:116411862	chr7:116411882	TAGCCGTCTTTAACAAGCTCT	-
MET	intron13	c.2888-37_2888-20del	chr7:116411866	chr7:116411883	CGTCTTTAACAAGCTCTT	-
MET	intron13	c.2888-36_2888-12del	chr7:116411866	chr7:116411890	CGTCTTTAACAAGCTCTTTCTTTCT	-
MET	intron13	c.2888-36_2888-29del	chr7:116411867	chr7:116411874	GTCTTTAA	-
MET	intron13	c.2888-36_2888-21del	chr7:116411867	chr7:116411882	GTCTTTAACAAGCTCT	-
MET	intron13	c.2888-36_2888-2del	chr7:116411867	chr7:116411901	GTCTTTAACAAGCTCTTTCTTTCTCTCTGTTTTAA	-
MET	intron13	c.2888-35_2888-27del	chr7:116411868	chr7:116411876	TCTTTAACA	-
MET	intron13	c.2888-35_2888-20del	chr7:116411868	chr7:116411883	TCTTTAACAAGCTCTT	-
MET	exon14	c.2888-35_2888del	chr7:116411868	chr7:116411903	TCTTTAACAAGCTCTTTCTTTCTCTCTGTTTTAAGA	-
MET	intron13	c.2888-28_2888-2del	chr7:116411870	chr7:116411896	TTTAACAAGCTCTTTCTTTCTCTCTGT	-
MET	intron13	c.2888-31_2888-13del	chr7:116411871	chr7:116411889	TTAACAAGCTCTTTCTTTC	-
MET	intron13	c.2887-32_2967del	chr7:116411871	chr7:116411982	TTAACAAGCTCTTTCTTTCTCTCTGTTTTAAGATCTGGGCAGTGAATTAGTTCGCTACGATGCAAGAGTACACACTCCTCATTTGGATAGGCTTGTAAGTGCCCGAAGTGTA	-

MET	intron13	c.2888-31_2888-15del&insA	chr7:116411872	chr7:116411888	TAACAAGCTCTTTCTTT	A
MET	intron13	c.2888-31_2888-10del&insGTT	chr7:116411872	chr7:116411893	TAACAAGCTCTTTCTTTCTCTC	GTT
MET	intron13	c.2888-30_2888-3del	chr7:116411873	chr7:116411900	AACAAGCTCTTTCTTTCTCTCTGTTTTA	-
MET	intron13	c.2888-29_2888-10del	chr7:116411874	chr7:116411893	ACAAGCTCTTTCTTTCTCTC	-
MET	intron13	c.2888-27_2888-14del	chr7:116411875	chr7:116411888	CAAGCTCTTTCTTT	-
MET	intron13	c.2888-28_2888-14del&insA	chr7:116411875	chr7:116411889	CAAGCTCTTTCTTTC	A
MET	intron13	c.2888-28_2888-5del	chr7:116411875	chr7:116411898	CAAGCTCTTTCTTTCTCTCTGTTT	-
MET	intron13	c.2888-27_2888-16del	chr7:116411876	chr7:116411887	AAGCTCTTTCTT	-
MET	intron13	c.2888-27_2888-10del&insT	chr7:116411876	chr7:116411893	AAGCTCTTTCTTTCTCTC	T
MET	intron13	c.2888-24_2888-1del	chr7:116411876	chr7:116411899	AAGCTCTTTCTTTCTCTCTGTTTT	-
MET	exon14	c.2888-25_2893del	chr7:116411878	chr7:116411908	GCTCTTTCTTTCTCTCTGTTTTAAGATCTGG	-
MET	exon14	c.2888-23_2918del	chr7:116411878	chr7:116411931	GCTCTTTCTTTCTCTCTGTTTTAAGATCTGGGCAGTGAATTAGTTCGCTACGAT	-
MET	intron13	c.2888-24_2888-15del	chr7:116411879	chr7:116411888	CTCTTTCTTT	-
MET	intron13	c.2888-24_2888-13del	chr7:116411879	chr7:116411890	CTCTTTCTTTCT	-
MET	intron13	c.2888-22_2888-12del	chr7:116411881	chr7:116411891	CTTTCTTTCTC	-
MET	intron13	c.2888-22_2888-8del	chr7:116411881	chr7:116411895	CTTTCTTTCTCTCTG	-
MET	intron13	c.2888-21_2888-9del	chr7:116411882	chr7:116411894	TTTCTTTCTCTCT	-
MET	intron13	c.2888-18_2888-5del	chr7:116411882	chr7:116411895	TTTCTTTCTCTCTG	-
MET	intron13	c.2888-21_2888-7del	chr7:116411882	chr7:116411896	TTTCTTTCTCTCTGT	-
MET	intron13	c.2888-21_2888-6del	chr7:116411882	chr7:116411897	TTTCTTTCTCTCTGTT	-
MET	intron13	c.2888-20_2888-4del	chr7:116411882	chr7:116411898	TTTCTTTCTCTCTGTTT	-
MET	intron13	c.2888-21_2888-2del	chr7:116411882	chr7:116411901	TTTCTTTCTCTCTGTTTTAA	-
MET	intron13	c.2888-19_2888-9del	chr7:116411883	chr7:116411893	TTCTTTCTCTC	-
MET	exon14	c.2888-20_2888del	chr7:116411883	chr7:116411903	TTCTTTCTCTCTGTTTTAAGA	-
MET	exon14	c.2888-20_2913del	chr7:116411883	chr7:116411928	TTCTTTCTCTCTGTTTTAAGATCTGGGCAGTGAATTAGTTCGCTAC	-
MET	intron13	c.2888-19_2888-10del	chr7:116411884	chr7:116411893	TCTTTCTCTC	-
MET	exon14	c.2888-19_2921del	chr7:116411884	chr7:116411936	TCTTTCTCTCTGTTTTAAGATCTGGGCAGTGAATTAGTTCGCTACGATGCAAG	-

**MET**	**intron13**	**c.2888-18_2888-17insAGA**	**chr7:116411885**	**chr7:116411886**	**CT**	**AGA**
**MET**	**intron13**	**c.2888-17_2888-8del**	**chr7:116411886**	**chr7:116411895**	**TTTCTCTCTG**	**-**
**MET**	**intron13**	**c.2888-17_2888-7del**	**chr7:116411886**	**chr7:116411896**	**TTTCTCTCTGT**	**-**
**MET**	**intron13**	**c.2888-15_2888-4del**	**chr7:116411886**	**chr7:116411897**	**TTTCTCTCTGTT**	**-**
**MET**	**intron13**	**c.2888-16_2888-4del**	**chr7:116411886**	**chr7:116411898**	**TTTCTCTCTGTTT**	**-**
**MET**	**intron13**	**c.2888-16_2888-4del&insC**	**chr7:116411887**	**chr7:116411899**	**TTCTCTCTGTTTT**	**C**
**MET**	**exon14**	**c.2888-15_2893del**	**chr7:116411888**	**chr7:116411908**	**TCTCTCTGTTTTAAGATCTGG**	**-**
**MET**	**exon14**	**c.2888-8_2924del&insTAAA**	**chr7:116411895**	**chr7:116411939**	**GTTTTAAGATCTGGGCAGTGAATTAGTTCGCTACGATGCAAGAGT**	**-**
**MET**	**exon14**	**c.2888-4_2910del**	**chr7:116411899**	**chr7:116411925**	**TAAGATCTGGGCAGTGAATTAGTTCGC**	**-**
**MET**	**intron13**	**c.G2888-1T**	**chr7:116411902**	**chr7:116411902**	**G**	**T**
**MET**	**exon14**	**c.2930_2932delCTC**	**chr7:116411945**	**chr7:116411947**	**CTC**	**-**
**MET**	**exon14**	**c.2995_3024del**	**chr7:116412010**	**chr7:116412039**	**GAATCTGTAGACTACCGAGCTACTTTTCCA**	**-**
**MET**	**exon14**	**c.2998_3028+6del**	**chr7:116412013**	**chr7:116412049**	**TCTGTAGACTACCGAGCTACTTTTCCAGAAGGTATAT**	**-**
**MET**	**exon14**	**c.3004_3028+17del**	**chr7:116412019**	**chr7:116412060**	**GACTACCGAGCTACTTTTCCAGAAGGTATATTTCAGTTTATT**	**-**
**MET**	**exon14**	**c.3005_3007del**	**chr7:116412020**	**chr7:116412022**	**ACT**	**-**
**MET**	**exon14**	**c.3007_3028+13del**	**chr7:116412022**	**chr7:116412056**	**TACCGAGCTACTTTTCCAGAAGGTATATTTCAGTT**	**-**
**MET**	**exon14**	**c.3012_3023del**	**chr7:116412027**	**chr7:116412038**	**AGCTACTTTTCC**	**-**
**MET**	**exon14**	**c.3012_3028+4del**	**chr7:116412027**	**chr7:116412047**	**AGCTACTTTTCCAGAAGGTAT**	**-**
**MET**	**exon14**	**c.3015_3028+3del**	**chr7:116412030**	**chr7:116412046**	**TACTTTTCCAGAAGGTA**	**-**
**MET**	**exon14**	**c.3018_3028+5del**	**chr7:116412033**	**chr7:116412048**	**TTTTCCAGAAGGTATA**	**-**
**MET**	**exon14**	**c.3019_3028+3del**	**chr7:116412034**	**chr7:116412046**	**TTTCCAGAAGGTA**	**-**
**MET**	**exon14**	**c.3019_3028+5del**	**chr7:116412034**	**chr7:116412048**	**TTTCCAGAAGGTATA**	**-**
**MET**	**exon14**	**c.3023_3028+13del**	**chr7:116412038**	**chr7:116412056**	**CAGAAGGTATATTTCAGTT**	**-**
**MET**	**exon14**	**c.3024_3028+14del**	**chr7:116412039**	**chr7:116412057**	**AGAAGGTATATTTCAGTTT**	**-**
**MET**	**exon14**	**c.3025_3028+2del**	**chr7:116412040**	**chr7:116412045**	**GAAGGT**	**-**
**MET**	**exon14**	**c.3028G>C(p.D1010H)**	**chr7:116412043**	**chr7:116412043**	**G**	**C**
**MET**	**intron14**	**c.G3028+1A**	**chr7:116412044**	**chr7:116412044**	**G**	**A**
**MET**	**intron14**	**c.3028+1_3028+4del**	**chr7:116412044**	**chr7:116412047**	**GTAT**	**-**
**MET**	**intron14**	**c.T3028+2C**	**chr7:116412045**	**chr7:116412045**	**T**	**C**
**MET**	**intron14**	**c.A3028+3T**	**chr7:116412046**	**chr7:116412046**	**A**	**T**

**MET**	**exon14**	**c.2887+95_3028+53del**	**chr7:116411803**	**chr7:116412096**	**TGTAGTCCATAAAACCCATGAGTTCTGGGCACTGGGTCAAAGTC<br>TCCTGGGGCCCATGATAGCCGTCTTTAACAAGCTCTTTCTTTCTC<br>TCTGTTTTAAGATCTGGGCAGTGAATTAGTTCGCTACGATGCAA<br>GAGTACACACTCCTCATTTGGATAGGCTTGTAAGTGCCCGAAGT<br>GTAAGCCCAACTACAGAAATGGTTTCAAATGAATCTGTAGACTAC<br>CGAGCTACTTTTCCAGAAGGTATATTTCAGTTTATTGTTCTGAGA<br>AATACCTATACATATACCTCAGTGGGT**	**-**
**MET**	**exon14**	**c.2888-20_3028+1133del**	**chr7:116411876**	**chr7:116413169**	**AAGCTCTTTCTTTCTCTCTGTTTTAAGATCTGGGCAGTGAATTAG<br>TTCGCTACGATGCAAGAGTACACACTCCTCATTTGGATAGGCTT<br>GTAAGTGCCCGAAGTGTAAGCCCAACTACAGAAATGGTTTCAAA<br>TGAATCTGTAGACTACCGAGCTACTTTTCCAGAAGGTATATTTCA<br>GTTTATTGTTCTGAGAAATACCTATACATATACCTCAGTGGGTTG<br>TGACATTGTTGTTTATTTTTGGTTTTGCATTTATATTTTTATAAAA<br>ACCTAAAGGAAGTATTTACCTCTGCCAAGTAAGTATTTGACACAA<br>AATTACATGGCTCTTAATTTTAAAAGAACCCATGTATATATTACAT<br>TATGATTTTAGAGTCCATAAGCTCTCATTTCACAAAAAGGTTAAT<br>TTGAGCAAAAGTAATTTGTTTATCATCTAAGTGCAATAGTAAGAA<br>ATTGCGAAGCTCTCTTTTACAATCCAGGAAGAGTTAAGTTACAAA<br>ATATACTTATTTAAATGTAAGTTGGAACTGCTACATTTTTTACCTG<br>TTGAAGCCCAAACATTGAAATTATACTGTTAGTAATTCTTCGAAG<br>TGTTTTCAATGAACTGTTAGTACACAGCCTTTTTCCCACCATATT<br>CTAGGACTTGAATGTATTTTGAGACTTAGCCAAGGAAAACCTTC<br>AATTATGCCATGAAAAAAAGGAGGGGTCAATATCATCAGCTTTGT<br>AAAACACTATGCCTAGTAATGTTCAGGTTAATCAGAGTTTTCATG<br>TTGTTTTATTTAAATCTCCTGGTAAAAGCAAAAGGTCTGTATTGT<br>ATCAGCTCCATTATCTTTAGAAGTTACAGGATGTGAGTCAAGTAC<br>AAGCATTTCCTTGGTTGAATATTTACCATTGGACAAATAAAATGA<br>GTCACAGATCATTGAGGATACTGGAAAAGTTAGAAGTTGCTCAT<br>CCAAACAAGTTCAAGAGCAATGAAGCACTTAACATTTTAACATTT<br>TCAACACTTACTACCTCTTATGTTTTGAAGTTTATGTTATTTCTAT<br>GGAGATACACATAGTAAACATTGTCTTTGCCCTGATTCCATTCAC<br>CTTTAAAAATCCATTCGTTTAACCGTGTGGAAAAATCAAACCTAG<br>TTTATTGTTTTGAAATTTAGATCTATTTAGTATTTTATGTGCACATT<br>TAGTGCATCTATTTAGTATTTTATATGCACATTTCATAGTTCTAATC<br>TGAGATCATTAAAATTTACAAATTTTCTTTGAAAAAAAAACTTAC<br>CTAATCTTCTTTGAACCTCCTTACTCACCA**	**-**
**MET**	**exon14**	**c.3010_3028+1318del**	**chr7:116412025**	**chr7:116413361**	**CGAGCTACTTTTCCAGAAGGTATATTTCAGTTTATTGTTCTGAGA<br>AATACCTATACATATACCTCAGTGGGTTGTGACATTGTTGTTTATT<br>TTTGGTTTTGCATTTATATTTTTATAAAAACCTAAAGGAAGTATTT**	**-**

					**ACCTCTGCCAAGTAAGTATTTGACACAAAATTACATGGCTCTTAA<br>TTTTAAAAGAACCCATGTATATATTACATTATGATTTTAGAGTCCA<br>TAAGCTCTCATTTCACAAAAAGGTTAATTTGAGCAAAAGTAATTT<br>GTTTATCATCTAAGTGCAATAGTAAGAAATTGCGAAGCTCTCTTT<br>TACAATCCAGGAAGAGTTAAGTTACAAAATATACTTATTTAAATGT<br>AAGTTGGAACTGCTACATTTTTTACCTGTTGAAGCCCAAACATTG<br>AAATTATACTGTTAGTAATTCTTCGAAGTGTTTTCAATGAACTGT<br>TAGTACACAGCCTTTTTCCCACCATATTCTAGGACTTGAATGTAT<br>TTTGAGACTTAGCCAAGGAAAACCTTCAATTATGCCATGAAAAA<br>AAGGAGGGGTCAATATCATCAGCTTTGTAAAACACTATGCCTAGT<br>AATGTTCAGGTTAATCAGAGTTTTCATGTTGTTTTATTTAAATCTC<br>CTGGTAAAAGCAAAAGGTCTGTATTGTATCAGCTCCATTATCTTT<br>AGAAGTTACAGGATGTGAGTCAAGTACAAGCATTTCCTTGGTTG<br>AATATTTACCATTGGACAAATAAAATGAGTCACAGATCATTGAGG<br>ATACTGGAAAAGTTAGAAGTTGCTCATCCAAACAAGTTCAAGAG<br>CAATGAAGCACTTAACATTTTAACATTTTCAACACTTACTACCTC<br>TTATGTTTTGAAGTTTATGTTATTTCTATGGAGATACACATAGTAA<br>ACATTGTCTTTGCCCTGATTCCATTCACCTTTAAAAATCCATTCG<br>TTTAACCGTGTGGAAAAATCAAACCTAGTTTATTGTTTTGAAATT<br>TAGATCTATTTAGTATTTTATGTGCACATTTAGTGCATCTATTTAG<br>TATTTTATATGCACATTTCATAGTTCTAATCTGAGATCATTAAAAT<br>TTACAAATTTTCTTTGAAAAAAAAACTTACCTAATCTTCTTTGAA<br>CCTCCTTACTCACCAAAGCTCTGTCATCATTGCTAAGAAGGTTGA<br>GTTTCACACTCTTTTCTCCATTGAGCCTGCTCCTTGGAGACATG<br>AAAAGAAAACAGGTAAAAGAGGGTCATTTAGAGAGAATGAGAAA<br>ATAGGTGCACAGCCAAAACCTAATGAAGAGGCAACTGCAGAGCT<br>TTCCTCTCTACATCTGGTGGGGACAGCATT**	

Supplementary Table 2. Patients harbored two different *MET*ex14 alteration types.

**Num.**	**Gene**	**Alteration**	**Sample type**	**MAF**
**1**	***MET***	c.3012_3023del (p.1022_1026del)	Tissue	0.82
		c.G3028+1T		0.82
**2**	***MET***	c.2995_3024del (p.1017_1026del)	Tissue	0.2886
		c.A3028+3G		0.2886
**3**	***MET***	c.2888-20_2888-4del	Tissue	0.0123
		c.3028+2T>C		0.2022

Supplementary Table 3. The mutations identified in pre- and post-treatment samples from three patients treated by crizotinib.

Patient ID	Gene	Alteration	Pre-treatment		Post-treatment	
			**Sample type**	**MAF/CN ratio**	**Sample type**	**MAF/CN ratio**
**P5**	***MET***	c.3028+1G>C	Plasma	0.701	Metastatic site (Adrenal gland)	0.5967
	ATR	c.695T>G (p.I232R)		0.0888		--
	GNAQ	c.303C>A (p.Y101*)		0.0575		--
	TYMS	c.2T>C (p.M1?)		0.0191		--
	CYLD	c.2173G>T (p.V725F)		0.0175		--
	APC	c.2534G>A (p.R845H)		0.0112		--
	EPAS1	c.95A>G (p.E32G)		0.0066		--
	EPAS1	c.96G>T (p.E32D)		0.006		--
	FANCE	c.274_364dup (p.Q122Pfs*37)		0.0138		--
	TET2	c.4222G>A (p.G1408R)		--		0.125
	BRAF	c.1801A>G (p.K601E)		--		0.0108
	EGFR	CNV		--		3.05
	MET	CNV		1.85		--
	MDM2	CNV		7.81		5.07
	TERT	CNV		5.76		2.56
	FLT4	CNV		2.23		--
	KIT	CNV		1.9		--
	FGFR4	CNV		1.88		--
	PDGFRA	CNV		1.8		--
	MYC	CNV		1.77		--
**P12**	***TP53***	c.476C>T (p.A159V)	Tissue	0.1771	Plasma	0.1283
	MET	c.2888-88_2901del		0.2517		0.3568
	MET	c.3688T>A (p.Y1230N)		--		0.1861
**P14**	***MET***	c.2888-22_2888-8delCTTTCTTTCTCTCTG	Tissue	0.0433	Plasma	0.002
	MET	c.3682G>A (p.D1228N)		--		0.001

--: not detected. Mutation was shown as mutant allele frequency. Gene copy number gain was shown as relative fold change to normal control sample.
